# Quantifying the Miscibility of Biobased Resins in Binary Polyisoprene Systems

**DOI:** 10.1021/acspolymersau.6c00038

**Published:** 2026-06-01

**Authors:** Johannes Rochau, Jürgen E. K. Schawe, Jorge Lacayo-Pineda

**Affiliations:** † Institut für Anorganische Chemie, Leibniz Universität Hannover, 30167 Hannover, Germany; ‡ Research and Development, Continental Reifen Deutschland GmbH, 30419 Hannover, Germany; § Department of Materials, 27219 ETH Zurich, 8093 Zurich, Switzerland

**Keywords:** polymer resin composites, DSC, glass transition, miscibility, elastomers

## Abstract

The miscibility of polymer–resin systems is a key factor for processing behavior and performance of elastomer compounds, yet it is commonly assessed only indirectly. In this work, a quantitative approach for evaluating experimental characterization of the molecular interaction of amorphous polymer filler composites is introduced based on glass transition measurements by differential scanning calorimetry (DSC). Binary mixtures of isoprene rubber (IR) with three distinct biobased resins, a terpene resin, a rosin ester, and a maleic-modified rosin ester, were investigated over the entire composition range. The molecular interaction is expressed by an interaction parameter which reveals stronger repulsive interactions between IR and the rosin ester compared to the other resins of investigation. Mechanical investigation by torque measurements during mixing and transmission electron microscopy (TEM) indicates a correlation between the interaction parameter measured by DSC and the miscibility of the components of the composites.

## Graphical abstract

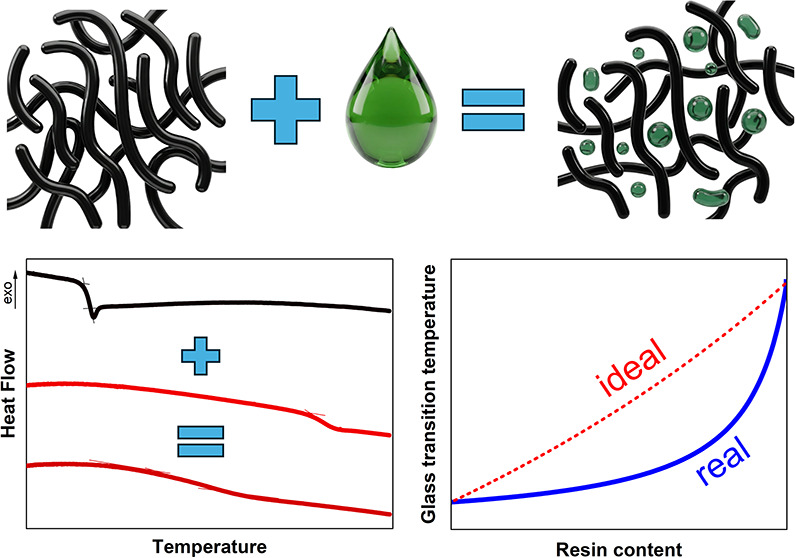

## 1 Introduction

The combination of polymers with functional additives is a growing trend in modern development of polymeric materials to tailor its properties.^1–3^ In high performance elastomeric polymers, a recent development strategy is the use of molecular designed resins as an additive.^4–6^ To form a homogeneous amorphous material with improved mechanical properties, it is required that resin and polymer are thermodynamically compatible.

Modern tire compounds are one of the largest industrial application of elastomer systems improved with resins, where they are used to enhance grip,^7^ rolling resistance^8^ and overall handling performance^9^ as well as processability.^10^ Their effect on the viscoelastic properties of elastomer matrices makes them indispensable for performance optimization in tread formulations.^11^ Despite their industrial relevance, the mixing behavior of resins and polymers is not understood in detail, and the known empirical rules are not generally applicable. The common approach for resin selection still follows a *trial-and-error* strategy, relying on experimental screening rather than predictive or systematic evaluation.

Traditional attempts to quantify compatibility, such as the use of solubility parameters (e.g., Hildebrand or Hansen parameters), provide only rough guidance.^12,13^ These parameters are derived from cohesive energy densities and assume ideal mixing behavior, neglecting complex molecular interactions, segmental dynamics, and specific interactions such as π–π or polar contributions.^14,15^ As a result, they are insufficient for describing the thermodynamic miscibility of polymer–resin systems in practical formulations.

In materials development, miscibility is therefore often inferred indirectly through macroscopic or processing-related observations. Mixer torque data^16^ and rheological behavior in the unvulcanized compound are used as qualitative indicators of phase compatibility.^17^ Mechanical properties such as the loss factor tan δ at 0 °C or at 60 °C are proposed as empirical predictors for miscibility.^16,18^ None of these approaches enable predictions on miscibility and provide a quantitative or thermodynamically based characterization. An alternative approach is the determination of the compositional dependence of the glass transition temperature, *T*_g_ by differential scanning calorimetry (DSC) and the evaluation using a modified Gordon-Taylor equation.^13,15,19^ While alternative thermodynamic models, such as the Flory–Huggins theory, are commonly applied to describe polymer miscibility, their applicability within the scope of this work is limited.^20^ The Flory–Huggins approach is based on a lattice (volume) theory and primarily accounts for combinatorial entropy and van der Waals-type interactions, which restricts its ability to capture more complex molecular interactions that are present in polymer–resin systems.^20^ Approaches that consider the chemical structure of the components are capable to describe complex variation in *T*_g_, however, the associated parameters cannot be unambiguously interpreted in terms of specific molecular interactions.^19^ This leads to the conclusion that, while complex and detailed models of molecular interactions provide a set of parameters that accurately describe the concentration dependence of *T*_g_, they do not offer deeper insights into the molecular interactions and are therefore limited in their applicability. The focus of this study lies in the development of a robust and experimentally accessible analysis method for practical compound design.

The appearance of a single or two separated glass transitions is often used to identify “miscible” and “immiscible” systems.^21,22^ However, this gives no information about the molecular interactions and is not a precise analytical framework to describe the extent of the miscibility of two components. In our opinion, a generalized, thermodynamically based method is required to evaluate miscibility on a quantitative and objective scale. Such a method should correlate with the classical mechanical indicators (e.g., mixing curves) and microscopical evidence (e.g., TEM),^23^ enabling a consistent and reproducible understanding of polymer–resin interactions.

Based on a standard thermodynamic approach, that has already proven its validity in carbohydrate-water systems,^24^ we introduce a method to characterize molecular interactions in binary systems using a model for the composition dependence of the glass transition temperature (*T*_g_). The model is applied for various polyisoprene-resin systems to quantify the miscibility, bridging the gap between thermodynamic analysis and conventional morphological or mechanical diagnostics. In order to introduce the method, only amorphous binary systems will be evaluated, as the interfacial physics of fillers add further complexity.^25^

## 2 Experimental Section

### 2.1 Materials

An isoprene rubber (IR) with 97% cis conformation was used. The molecular weight and glass transition temperature are *M*_w_ = 1300 kg/mol, *M*_n_ = 345 kg/mol and *T*_g_ = −64 °C. This IR grade was selected to closely match the molecular weight and linearity of industrially relevant natural rubber (NR), while offering a significantly lower compositional complexity, minimizing confounding effects. This allows for a more controlled investigation of polymer–resin interactions and the development of an analytical framework. The polymer was mixed with three commercially available, biobased resins, the first being an oligomer resin based on a terpene monomer (*M*_w, oligomer_ = 1170 g/mol) SYLVATRAXX 8115 (Kraton Chemical B.V., Almere, The Netherlands), predominantly consisting of α-pinene with minor parts of β-pinene and dipentene/limonene, the second resin (SYLVATRAXX 2097 (Kraton Chemical B.V., Almere, The Netherlands)) consisting of a rosin ester (*M*_w_ = 1210 g/mol) whereas the third resin is based on a maleic-modified rosin ester (*M*_w_ = 1820 g/mol) in analogy to Prakoso et al.^26^ Structural formulas of IR and the resins are given in Figure 1. Toluene (VWR, Avantor performance material LLC, Center Valley, USA) was used as a solvent for liquid mixing without further purification.

**Figure 1:**
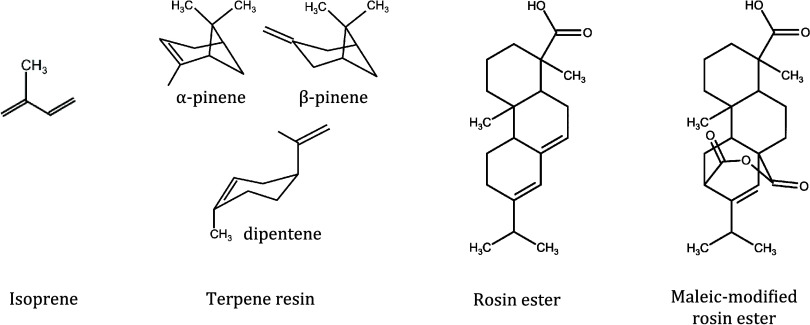
Molecular structures of the isoprene monomer, resin monomers and biobased building block units used by the resins in this work.

Based on the apparent molecular similarity of the chemical structure, a systematic trend in miscibility with isoprene rubber is anticipated for the investigated polymer-resin composites: The terpene resin exhibits high miscibility due to its close chemical similarity to the IR backbone, favoring compatible interactions. The rosin ester is presumed to display reduced compatibility, as the presence of polar carboxylic functionalities introduces unfavorable interactions with the nonpolar polymer matrix. The modified rosin ester should have the lowest degree of miscibility, as its increased polarity combined with a chemically reinforced, more rigid molecular structure further limits conformational adaptability and suppresses favorable mixing with IR.

### 2.2 Sample Preparation

Binary mixtures of IR and each resin were prepared at resin concentrations of 0.0, 9.1, 16.7, 20.0, 28.6, 40.0, 60.0, and 80.0 wt %. To avoid any influence on polymer–resin miscibility, no vulcanization agents were added during mixing, since any resin affinity to vulcanization agents could reduce the effective resin content that interacts with the polymer, consequently resulting in a dilution effect.

Two preparation methods were employed depending on resin content. For low resin concentrations up to 28.6 wt %, conventional mechanical mixing was conducted using a 300 mL internal mixer (Haake Rheomix, Thermo Fisher Scientific, Waltham, MA, USA) at approximately 140 °C for 4 min.

Mixtures with resin contents from 20 wt % to 80 wt % were prepared using a liquid mixing technique. This was necessary because, with increasing resin concentration, the compounds became too soft to be effectively processed in an internal mixer, preventing sufficient force input and, consequently, efficient mixing. The polymer and resin were dispersed in the required ratios and dissolved in 5 mL of toluene. The mixtures were left to equilibrate for 72 h, after which the solvent was removed by rotary evaporation under vacuum at 70 °C. The absence of residual toluene in the liquid mixed samples was confirmed by FTIR spectroscopy. A list of all prepared samples is shown in Table S1.

### 2.3 Analytical Methods

#### 2.3.1 Differential Scanning Calorimetry (DSC)

DSC measurements were performed at a scanning rate of 5 K/min between −120 and 120 °C under nitrogen flow of 50 mL/min using a DSC3 (Mettler-Toledo, Nänikon, Switzerland) with liquid nitrogen cooling. To erase possible processing histories, all samples underwent an identical annealing and cooling cycle before the measurement consisting of a first heating cycle from 25 to 120 °C with 5 K/min, followed by cooling to −120 °C with −5 K/min.

For the preparation of specimens, the compounds were pressed at 4.5 bar and 100 °C to produce thin sheets with a thickness of 0.5 mm. Samples with a mass between 5 and 10 mg were transferred into the DSC crucible (Al 40 μL).

#### 2.3.2 Transmission Electron Microscopy (TEM)

TEM measurements were performed on a JEM 1400 (Jeol, Tokyo, Japan) at an acceleration voltage of 100 kV. The 60 nm thin specimens were prepared at −60 °C with a Leica cryo-ultramicrotome EM UC6/EM FC6 (Leica Microsystems, Wetzlar, Germany) and placed on a 300-mesh gold TEM-grid.

In order to prepare thin cuts for TEM analysis, the samples were vulcanized by using the Ebonite method.^27^ Small sections of the compounds were heated to 140 °C for 2 h in a (90:5:5 by weight) mixture of sulfur, N,N-Dicyclohexylbenzothiazole-2-sulfenamide (DCBS) (Merck KGaA, Darmstadt, Germany) and zinc stearate (Fisher Scientific GmbH, Schwerte, Germany). Ultramicrotomic cuts of samples containing more than 20 wt % resin exhibit insufficient stability on the TEM-grid and cannot be examined.

## 3 Glass Transition Temperature of Compatible Blends

One method for evaluating mixtures of the compounds A and B is the Gordon–Taylor equation^28^ which yields the general equation

1 $$T_{g}=\frac{w_{A}T_{g,A}+k_{0}w_{B}T_{g,B}}{w_{A}+{k_{0}w}_{B}}$$

in which *T*_g,i_ and *w*_i_ are the glass transition temperatures and the mass content in weight percent, wt % of the *i*th pure component (i = {A, B}). The parameter *k*_0_ = Δ*c*_B_/Δ*c*_A_ is the ratio of the heat capacity change at the glass transition temperatures of the components A and B. Here, A is the component with the lower *T*_g_ which in this work is IR. This approach is just applicable for athermal mixtures without specific molecular interactions.^13^ For many different materials it was shown that (eq 1) also describes the composition dependence of the glass transition temperature by substitution of *k*_0_ by a constant *k*, which characterizes the molecular interaction.^13,29^ Based on the one-parameter Margules equation^30^

2 $$\Delta h_{e}=w_{A}w_{B}a$$

­(Δ*h*_e_ is the excess enthalpy of the mixture and *a* the interaction parameter), the new parameter *k* can be expressed by

3 $$k=\frac{k_{0}+b}{1-b}$$

The new parameter *b* is related to the interaction parameter *a*

4 $$a=2b{\Delta c}_{A}\left(T_{g,A}-T_{g,B}\right)$$

The derivation of these relations is given in the Appendix. This equation allows an easy determination of the interaction parameter by measurement of the composition dependence of the glass transition.

## 4 Results and Discussion

Heat flow curves measured by DSC are shown in Figure 2 (a)–(c). The tangents indicating the glass transitions are added. All samples exhibit a single glass transition. The glass transition temperature *T*_g_ and the width of the glass transition depend on the resin type and concentration. For each pure component–IR and the respective resins–the specific heat capacity steps at the glass transition temperature, Δ*c*, were determined and used for the calculation of the parameter *k*_0_. Using the measured *T*_g_ dependence on the composition (Figure 2(d)), the Gordon–Taylor equation was fitted to obtain the effective Gordon–Taylor constant *k*. The interaction parameter *a* is determined according to eqs 2 and ([Disp-formula eq3]). The values of Δ*c*, *k*_0_, *k* and *a* are listed in Table 1. The difference between *k* and *k*_0_ indicates the presence of specific molecular interactions for all binary systems as expected for thermal mixtures. The interaction parameter *a* indicates a similar molecular interaction of the terpene resin and the modified rosin ester with isoprene rubber, whereas this interaction is significantly larger for IR with rosin ester. The derivation of parameter *a* is based on the molecular interaction energy ε, as outlined in Appendix (eq A17). The sign of ε reflects the nature of the molecular interactions: ε < 0 corresponds to attractive interactions, whereas ε > 0 indicates repulsive interactions.^30^ In the present case, ε assumes positive values, demonstrating the predominance of repulsive polymer-resin interactions. Such repulsive contributions thermodynamically disfavor mixing, promote local phase separation between the rubber matrix and the resin and consequently reduce overall miscibility. According to the data, the rosin ester would be expected to show a reduced miscibility with the IR matrix as the repulsive interactions between polymer and resin are stronger.

**Figure 2:**
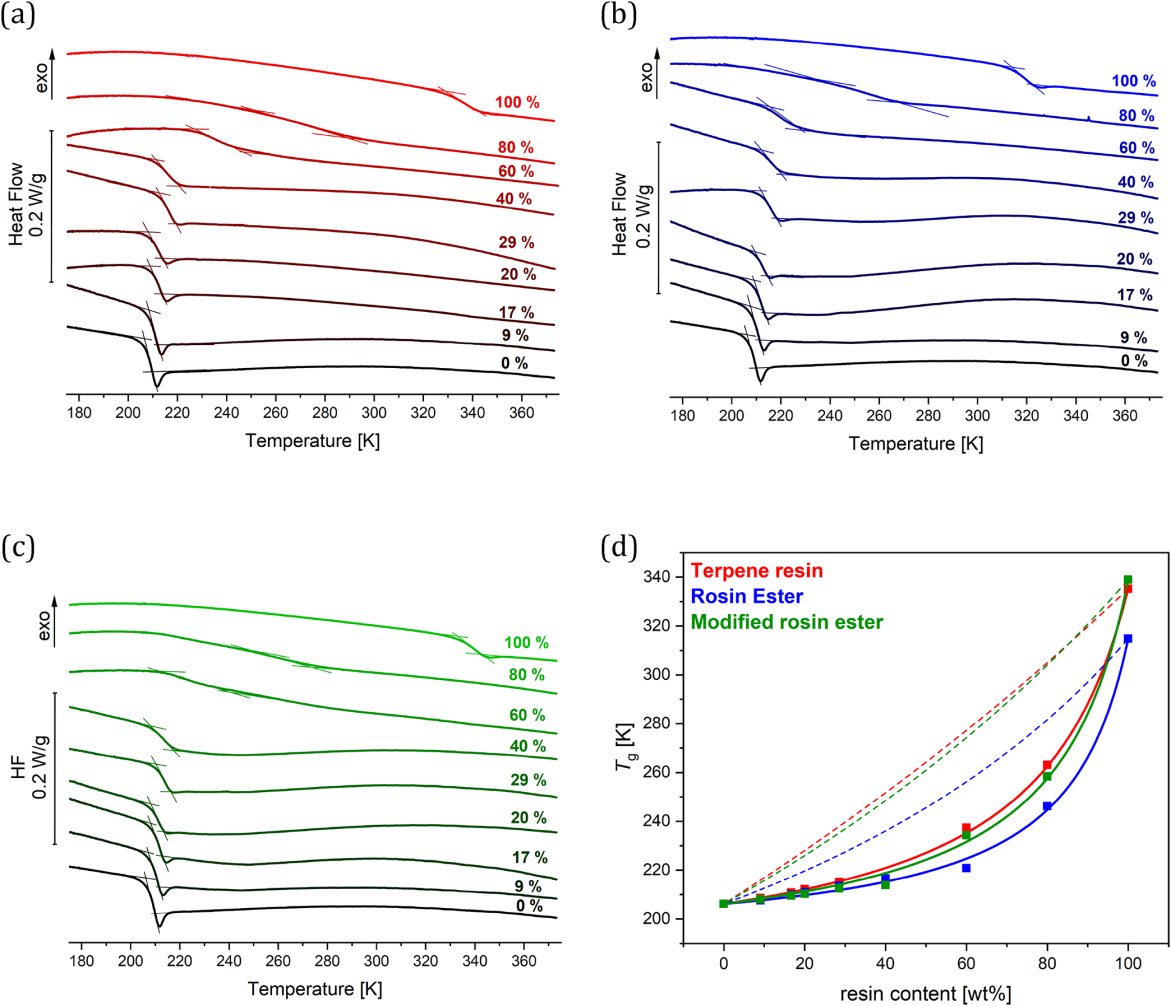
Heat flow curves with indicated *T*_g_ evaluation of (a) terpene resin, (b) rosin ester, (c) modified rosin ester in IR at different concentrations. (d) resulting Gordon–Taylor fits. Dotted lines represent the theoretical *T*_g_ behavior based on the ideal *k*_0_ parameter.

**Table 1:** Glass Transition Temperature *T*_g_ and the Intensity of the Glass Transition Δ*c* of the Pure Components as well as the Gordon Taylor Parameter *k*_0_, the Fitted Value *k* and the Interaction Parameter *a* of the Composites

component	*T*_g_ [K]	Δ*c* [J g^–1^ K^–1^]	*k*_0_	*k*	*a* [J g^–1^]
IR	206.7	0.54	-	-	-
terpene resin	335.7	0.25	0.46	0.19	25.1
rosin ester	314.4	0.31	0.57	0.14	32.2
modified rosin ester	338.9	0.24	0.45	0.16	28.8

To validate these thermodynamic findings through independent methods, mixer torque curves were recorded for compounds containing 28.6 wt % resin. The torque–time profiles (Figure 3) show a clear differentiation between the resin systems. Blends containing terpene resin (Figure 3(a)) and modified rosin ester (Figure 3(c)) display an immediate and steep increase in torque *S* after the initial mixing step within the first ∼50 s. The increase in torque is the consequence of the rapid viscosity build-up caused by the efficient incorporation of the resin into the polymer matrix. This behavior is characteristic of systems in which the resin disperses uniformly into the rubber phase. In contrast, the rosin ester compound (Figure 3(b)) showed a slower increase in torque, so that the rotor speed *n* had to be increased twice. After increasing the rotor speed, the torque, *S*, slowly rose to similar levels as the other two resin compounds, indicating a different incorporation behavior.

**Figure 3:**
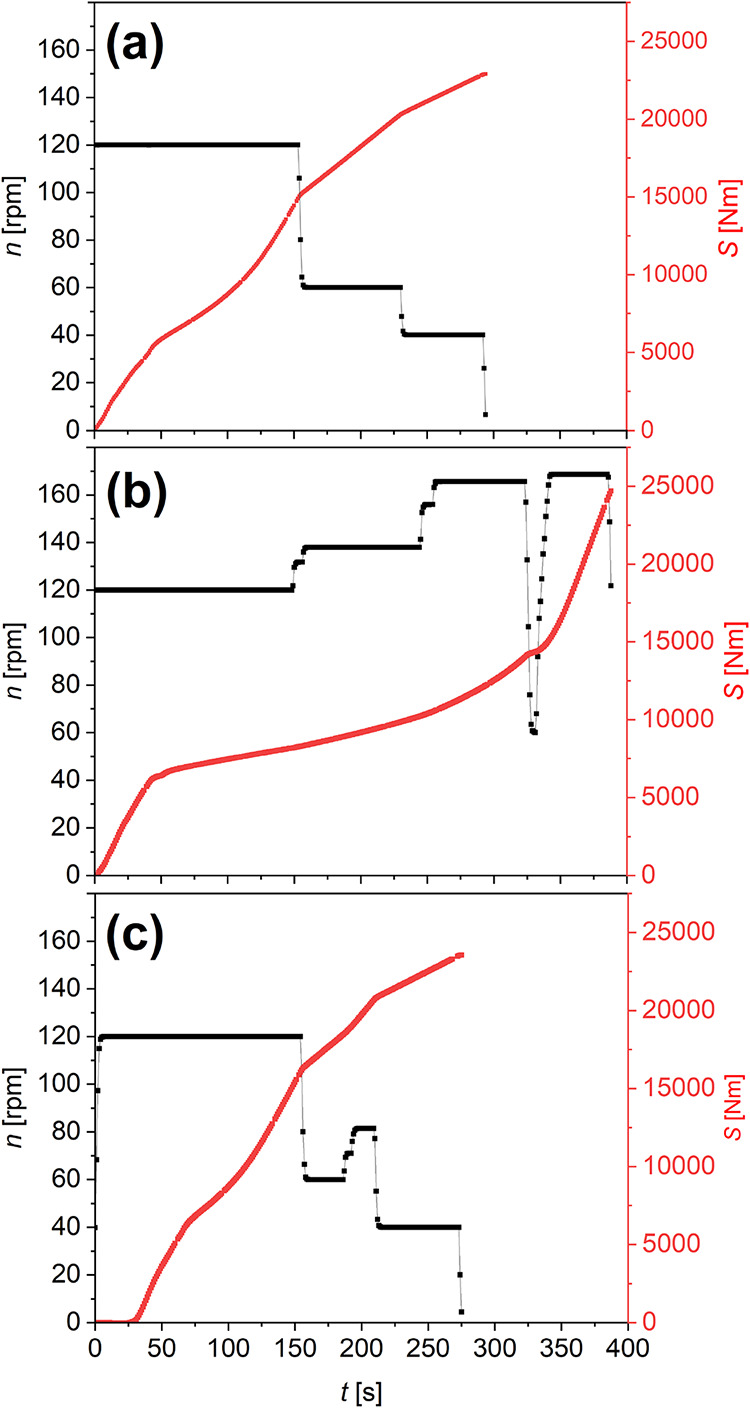
Torque-time profiles of IR with 28.6 wt % of (a) terpene resin, (b) rosin ester and (c) modified rosin ester.

An additional, independent method to verify the DSC-derived miscibility assessment is transmission electron microscopy (TEM). In order to obtain sufficiently rigid samples for ultrathin sectioning by cryo-ultramicrotomy the polymer–resin composites needed to be vulcanized first. To avoid any influence of a vulcanization system on the intrinsic miscibility of the binary IR–resin blends, the compounds were first mixed without curatives. Vulcanization was subsequently carried out using the Ebonite method.^27^

Figure 4 shows representative micrographs of the three IR-resin formulations with 20 wt % resin content. The blend containing terpene resin (Figure 4a) exhibits a homogeneous morphology without discernible phase domains, indicating uniform dispersion of the resin within the IR matrix. An analogous morphology is observed for the compound containing the modified rosin ester (Figure 4c), which is consistent with the favorable miscibility indicated by the thermodynamic analysis. In contrast, the compound prepared with rosin ester (Figure 4b) shows phase-separated particles approximately 50–100 nm in diameter distributed throughout the matrix. These domains appear with diffuse rather than sharp interfaces, suggesting partial—though significantly reduced—miscibility rather than complete immiscibility. The absence of a well-defined interface indicates that the resin still interacts to some extent with the polymer but fails to achieve full molecular-level mixing.

**Figure 4:**
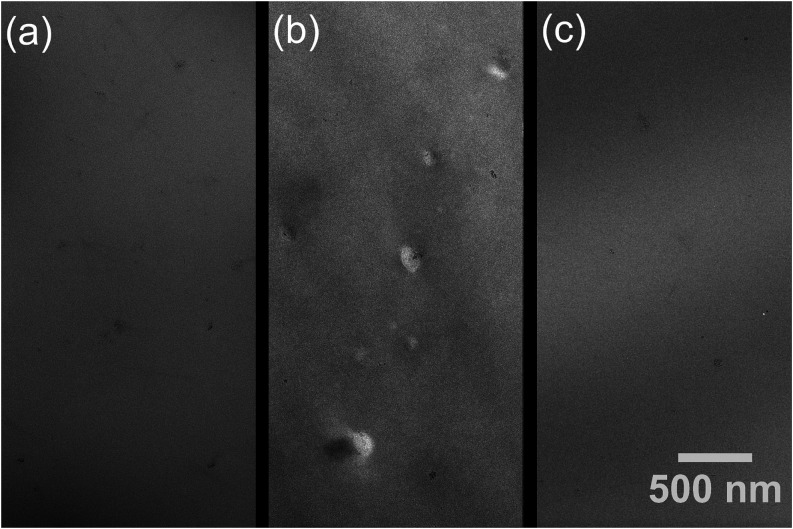
TEM micrograph of IR and different resins. (a) terpene resin, (b) rosin ester and (c) modified rosin ester.

All samples exhibit occasional small artifacts, visible as little dark spots, which likely arise from unavoidable contamination or debris introduced during internal-mixer processing. These do not affect the interpretation of the miscibility behavior.

The molecular reason for the reduced miscibility of the rosin ester in IR remains unclear and is not primarily the focus of this investigation. The proposed method allows for the quantification of miscibility, while the molecular origin would require further investigation by spectroscopic or computational methods.

## 5 Conclusion

In this study, the miscibility of three structurally distinct resins with isoprene rubber was systematically investigated using a DSC-based evaluation approach supported by mechanical and microscopical methods. The initial expectation based on the comparison of the molecular structure that the terpene resin would be highly miscible, the rosin ester moderately miscible, and the modified rosin ester poorly miscible with IR—was clearly disproven. This finding demonstrates that simple chemical similarity arguments are insufficient to reliably predict miscibility in polymer–resin systems and a new method of quantification is needed.

The phase diagram of the glass transition temperature was measured and a molecular interaction parameter *a* was evaluated using a modified Gordon–Taylor approach. This parameter reflects molecular-level interactions and reveals significantly stronger repulsive interactions between isoprene rubber and the rosin ester compared to the terpene resin and the modified rosin ester. The thermodynamic ranking derived from DSC measurements is consistent with the observed processing behavior and microscopic analysis performed by TEM.

The presented approach establishes a new pathway for incorporating molecular interaction parameters into miscibility analysis of polymer resin systems based on calorimetric investigations.

The applicability of the proposed method has so far been validated for binary isoprene rubber–resin systems, establishing its reliability under well-defined and simplified conditions. Future work will extend this approach to other elastomer matrices, such as styrene–butadiene rubber, in order to assess its generality across technologically relevant polymer systems. In addition, further work will include the influence of reinforcing silica to evaluate how filler–polymer–resin interactions affect the derived thermodynamic parameters and to further broaden the applicability of the method toward realistic rubber compound formulations.

## Supplementary Material



## Notes

Derivation of the extended Gordon Taylor approach, a table of all investigated samples as well as further validation experiments (PDF)
