# Supplementary material for: Quantifying the Miscibility of Biobased Resins in Binary Polyisoprene Systems
Source: ACS Polym Au. 2026 Jun 1;6(4):1110–5. doi: 10.1021/acspolymersau.6c00038 (PMC13474347; doi:10.1021/acspolymersau.6c00038)
Supplement: Supplementary file 1 [file lg6c00038_si_001.pdf]

Supporting Information

Quantifying the Miscibility of Bio-based Resins in Binary Polyisoprene Systems

*Johannes Rochau**[1,2], *Jürgen E. K. Schawe*[3], *Jorge Lacayo-Pineda*[1,2]

1 – Institut für Anorganische Chemie, Leibniz Universität Hannover, 30167 Hannover, Germany
2 – Research and Development, Continental Reifen Deutschland GmbH, 30419 Hannover, Germany
3 – Department of Materials, ETH Zurich, 8093 Zurich, Switzerland

Content:

Derivation of extended Gordon Taylor approach

Table S1 of all investigated samples

Figure S1 and S2 with additional measurements for validation the sample preparation.

Derivation of extended Gordon-Taylor approach

The composition dependence of the enthalpy of the ideal athermal mixture in the supercooled liquid can be described by

$$h_{\mathrm{m,l}} \;=\; x_{\mathrm{A}} h_{\mathrm{A,m,l}} + x_{\mathrm{B}} h_{\mathrm{B,m,l}} \tag{A1}$$

where $h_{\mathrm{m,l}}$ is the molar enthalpy of the mixture in the liquid, $h_{\mathrm{i,m,l}}$ the related molar enthalpies of the pure component i (i = A, B) and x_{i} the respective mol fraction. For the glassy state follows

$$h_{\mathrm{m,g}} \;=\; x_{\mathrm{A}} h_{\mathrm{A,m,g}} + x_{\mathrm{B}} h_{\mathrm{B,m,g}}. \tag{A2}$$

As in the vitrification process during cooling, the glass transition occurs when the enthalpy of the liquid equals the enthalpy of the glass ($h_{\mathrm{m,l}} = h_{\mathrm{m,g}}$). The temperature dependence of the enthalpy of the pure components reads

$$h_{\mathrm{i,m,j}}(T) \;=\; h_{\mathrm{i,m,j}}(T_{\mathrm{g,i}}) + \int_{T_{\mathrm{g,i}}}^{T} c_{\mathrm{i,m,j}}(T)\,\mathrm{d}T \tag{A3}$$

where the subscript j describes the liquid phase (j=l) or the glassy phase (j=g), $T_{\mathrm{g,i}}$ is the glass transition temperature of the ith pure component and $c_{\mathrm{i,m,j}}$ the molar heat capacity of the jth phase of the ith component. In a good approximation, the temperature dependence of the heat capacity can be neglected. In this case, equation (A3) simplifies to

$$h_{\mathrm{i,m,j}}(T) \;=\; h_{\mathrm{i,m,j}}(T_{\mathrm{g,i}}) + c_{\mathrm{i,m,j}}(T - T_{\mathrm{g,i}}). \tag{A4}$$

At the glass transition temperature of the mixture, T_{g} is $h_{\mathrm{m,l}}(T_g) = h_{\mathrm{m,g}}(T_g)$. By combination of equation (A1) and (A2) follows

$$x_{\mathrm{A}} h_{\mathrm{A,m,l}}(T_{\mathrm{g}}) + x_{\mathrm{B}} h_{\mathrm{B,m,l}}(T_{\mathrm{g}}) \;=\; x_{\mathrm{A}} h_{\mathrm{A,m,g}}(T_{\mathrm{g}}) + x_{\mathrm{B}} h_{\mathrm{B,m,g}}(T_{\mathrm{g}}) \tag{A5}$$

For the case that thermal excess enthalpy h_{e} occurs, equation (A1) is extended to

$$h_{\mathrm{m,j}}(T) \;=\; x_{\mathrm{A}} h_{\mathrm{A,m,j}}(T) + x_{\mathrm{B}} h_{\mathrm{B,m,j}}(T) + h_{\mathrm{e,j}}(T). \tag{A6}$$

By using equation (A4) for the enthalpy of the pure components and under consideration of $h_{\mathrm{m,l}}(T_{\mathrm{g}}) = h_{\mathrm{m,g}}(T_{\mathrm{g}})$, equation (A5) reads

$$x_{\mathrm{A}} \Delta c_{\mathrm{A,m}}(T_{\mathrm{g}} - T_{\mathrm{g,A}}) + x_{\mathrm{B}} \Delta c_{\mathrm{B,m}}(T_{\mathrm{g}} - T_{\mathrm{g,B}}) + \Delta h_{\mathrm{e}}(T_{\mathrm{g}}) \;=\; 0 \tag{A7}$$

where $\Delta h_{\mathrm{e}} = h_{\mathrm{e,l}} - h_{\mathrm{e,g}}$. Rearrangement of equation (A7) yields

$$T_{\mathrm{g}} \;=\; \frac{x_{\mathrm{A}} T_{\mathrm{g,A}}\, \Delta c_{\mathrm{A,m}} + x_{\mathrm{B}} T_{\mathrm{g,B}} \left(\Delta c_{\mathrm{B,m}} + \frac{\Delta h_{\mathrm{e}}}{x_{\mathrm{B}}(T_{\mathrm{g}} - T_{\mathrm{g,B}})} \right)}{x_{\mathrm{A}} \Delta c_{\mathrm{A,m}} + x_{\mathrm{B}} \left(\Delta c_{\mathrm{B,m}} + \frac{\Delta h_{\mathrm{e}}}{x_{\mathrm{B}}(T_{\mathrm{g}} - T_{\mathrm{g,B}})} \right)} \tag{A8}$$

which can be expressed in a Gordon-Taylor structure with the introduction of a generalized parameter

$$k_{\mathrm{m}} = k_{\mathrm{m0}} + \frac{\Delta h_{\mathrm{e}}}{\Delta c_{\mathrm{A,m}} x_{\mathrm{B}} (T_{\mathrm{g}} - T_{\mathrm{g,B}})}, \tag{A9}$$

with the second term carrying the information about the molecular interaction during mixing.

From Equations (A8) and (A9) follows the modified Gordon-Taylor equation

$$T_{\mathrm{g}} \;=\; \frac{x_{\mathrm{A}}\, T_{\mathrm{g,A}} + k_{\mathrm{m}}\, x_{\mathrm{B}}\, T_{\mathrm{g,B}}}{x_{\mathrm{A}} + k_{\mathrm{m}}\, x_{\mathrm{B}}} \tag{A10}$$

In the next step we would express Δh_{e} in terms of molecular interaction to find finally a practical expression for k_{m}. For the description of molecular interactions, various models can be used. The simplest model is the one-parameter Margules equation, also known as Porter approach[25]

$$\Delta g_{\mathrm{e}} \;=\; x_{\mathrm{A}} x_{\mathrm{B}} A_{\mathrm{m}} \tag{A11}$$

where Δg_{e} is the excess Gibbs free energy and A_{m} characterizes the interaction between molecules A and B. From

$$\Delta h_{\mathrm{e}} = \Delta g_{\mathrm{e}} + T \left(\frac{\partial g_{\mathrm{e}}}{\partial T} \right)_{p,x} \tag{A12}$$

follows

$$\Delta h_{\mathrm{e}} = x_{\mathrm{A}} x_{\mathrm{B}} \left[A_{\mathrm{m}} + T \left(\frac{\partial A_{\mathrm{m}}}{\partial T} \right) \right]. \tag{A13}$$

The temperature dependence of A_{m} can be approximated by the first two terms of a Taylor series expansion

$$A_{\mathrm{m}}(T) = A_{\mathrm{m}}(T_{\mathrm{g,B}}) + \left. \frac{\partial A_{\mathrm{m}}}{\partial T} \right|_{T_{\mathrm{g,B}}} (T - T_{\mathrm{g,B}}). \tag{A14}$$

By insertion of equation (A14) into (A13) follows

$$\Delta h_{\mathrm{e}} = x_{\mathrm{A}} x_{\mathrm{B}} \left[A_{\mathrm{m}}(T_{\mathrm{g,B}}) + T_{\mathrm{g,B}} \left. \frac{\partial A_{\mathrm{m}}}{\partial T} \right|_{T_{\mathrm{g,B}}} \right]. \tag{A15}$$

The term in brackets is constant, consequently equation (A15) can be simplified to

$$\Delta h_e = x_A x_B\, a_m. \quad (A16)$$

The simplest approach for a two-component system is

$$a_m = N_A\, z\, \varepsilon \quad (A17)$$

where N_A is the Avogadro constant, z is the number of molecules with which a molecule interacts on average and ε is the interaction energy. This is the difference between the interaction energy of A-B with the average of the interaction energies of A-A and B-B. If $\varepsilon < 0$, molecules will prefer to be surrounded by molecules of the opposite type. In the opposite case, the number of interactions of the type A-B is less than in the random solution. In other words, $\varepsilon < 0$ results in attractive interactions, $\varepsilon > 0$ in repulsive interactions[25].

By the insertion of equation (A16), the excess enthalpy term in (A9) reads

$$\frac{\Delta h_e}{\Delta c_{A,m}\, x_B (T_g - T_{g,B})} = \frac{x_A\, a_m}{\Delta c_{A,m}\, (T_g - T_{g,B})} \approx \frac{a_m}{2\Delta c_{A,m} (T_g - T_{g,B})} \quad (A18)$$

The approximation considers that the excess enthalpy is negligible for small and large values of x_A and the contribution is at a maximum at $x_A \approx 0.5$.

To describe the dependence of T_g at $x_A = 0.5$, equation (A8) with the generalized parameter k_m is used, yielding

$$\frac{a_m}{2\Delta c_{A,m}\, (T_g - T_{g,B})} = \frac{a_m}{2\Delta c_{A,m} \left(\frac{T_{g,A} + k_m\, T_{g,B}}{1 + k_m} - T_{g,B} \right)}$$

$$= \frac{a_m (1 + k_m)}{2\Delta c_{A,m}\, (T_{g,A} - T_{g,B})} = b_m (1 + k_m) \quad (A19)$$

Inserting $b_m(1 + k_m)$ into equation (A9) leads to

$$k_m = k_{m0} + b_m (1 + k_m). \quad (A20)$$

By transformation from molar properties to weight content, the generalized GT parameter reads

$$k = \frac{k_0 + b}{1 - b} \quad (A21)$$

with

$$b = \frac{a}{2\Delta c_A\, (T_{g,A} - T_{g,B})} \quad (A22)$$

and

$$a = \frac{2\, a_m}{M_A + M_B}. \quad (A23)$$

where M_A and M_B are the molecular weights of the polymer and the resin components.

Table S1: List of prepared samples and employed mixing technique. Values in brackets are informative only, since mechanical mixing is typically set up using phr. The different mixing methods include an overlap and an identical concentration at 28.6 wt% corresponding to 40 phr terpene resin concentration.

Sample	$w_{Polymer}$ [wt%]	$w_{Terpene\ resin}$ [wt%]	$w_{Rosin\ ester}$ [wt%]	$w_{mod.\ rosin\ ester}$ [wt%]	Mixing technique
001	100.0	0	-	-	Mechanical mixing
002	90.9	9.1 (10 phr)	-	-	Mechanical mixing
003	83.3	16.7 (20 phr)	-	-	Mechanical mixing
004	80.0	20.0 (25 phr)	-	-	Liquid mixing
005	71.4	28.6 (40 phr)	-	-	Mechanical mixing
006	71.4	28.6 (40 phr)	-	-	Liquid mixing
007	60.0	40.0	-	-	Liquid mixing
008	40.0	60.0	-	-	Liquid mixing
009	20.0	80.0	-	-	Liquid mixing
010	0	100	-	-	-
011	90.9	-	9.1 (10 phr)	-	Mechanical mixing
012	83.3	-	16.7 (20 phr)	-	Mechanical mixing
013	80.0	-	20.0 (25 phr)	-	Liquid mixing
014	71.4	-	28.6 (40 phr)	-	Mechanical mixing
015	60.0	-	40.0	-	Liquid mixing
016	40.0	-	60.0	-	Liquid mixing
017	20.0	-	80.0	-	Liquid mixing
018	0	-	100	-	-
019	90.9	-	-	9.1 (10 phr)	Mechanical mixing
020	83.3	-	-	16.7 (20 phr)	Mechanical mixing
021	80.0	-	-	20.0 (25 phr)	Liquid mixing
022	71.4	-	-	28.6 (40 phr)	Mechanical mixing
023	60.0	-	-	40.0	Liquid mixing
024	40.0	-	-	60.0	Liquid mixing
025	20.0	-	-	80.0	Liquid mixing
027	0	-	-	100	-

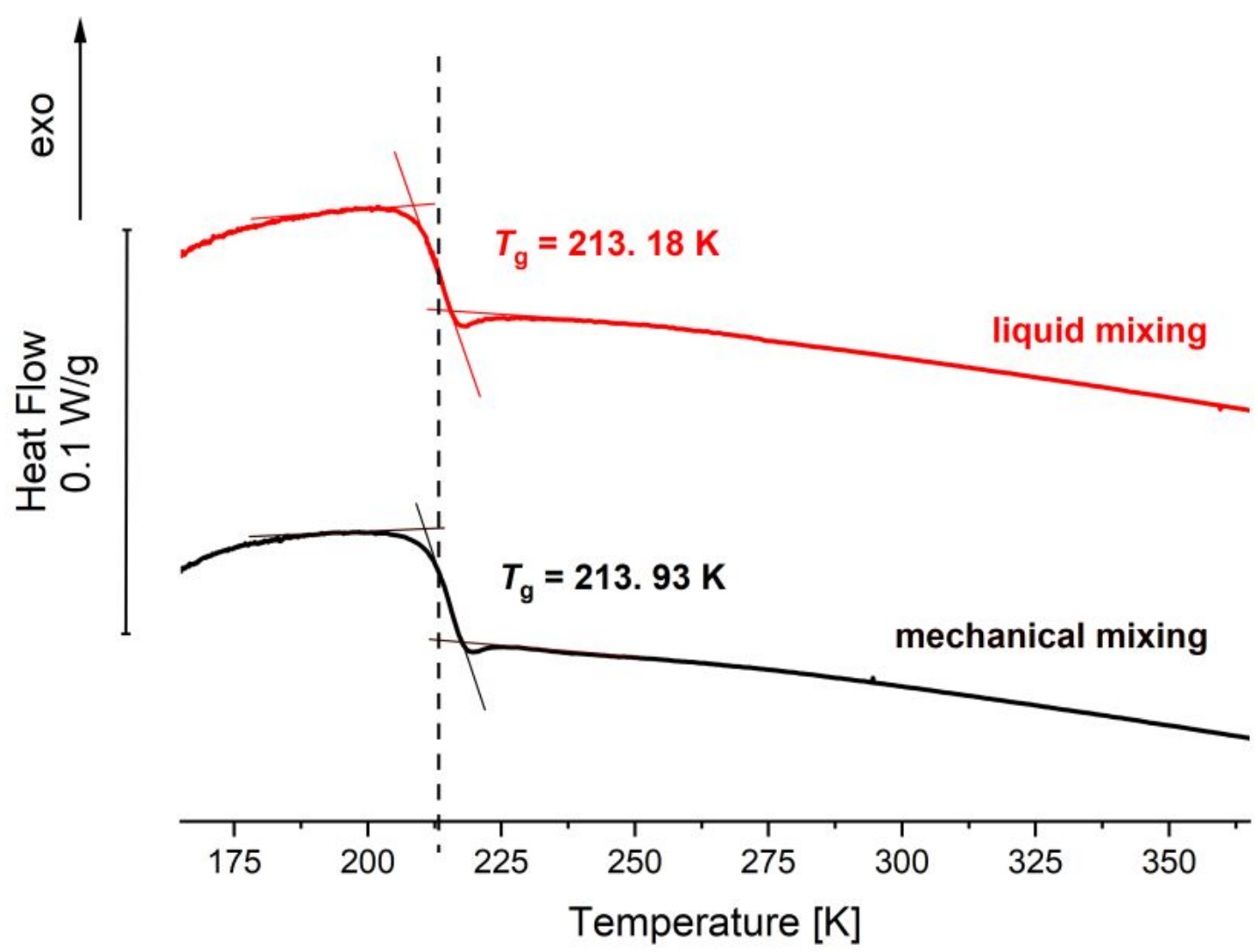

Figure S1: Heat flow curves of samples with 28.6 wt% terpene resin in IR prepared by mechanical mixing and liquid mixing. Both curves show an identical glass transition behavior.

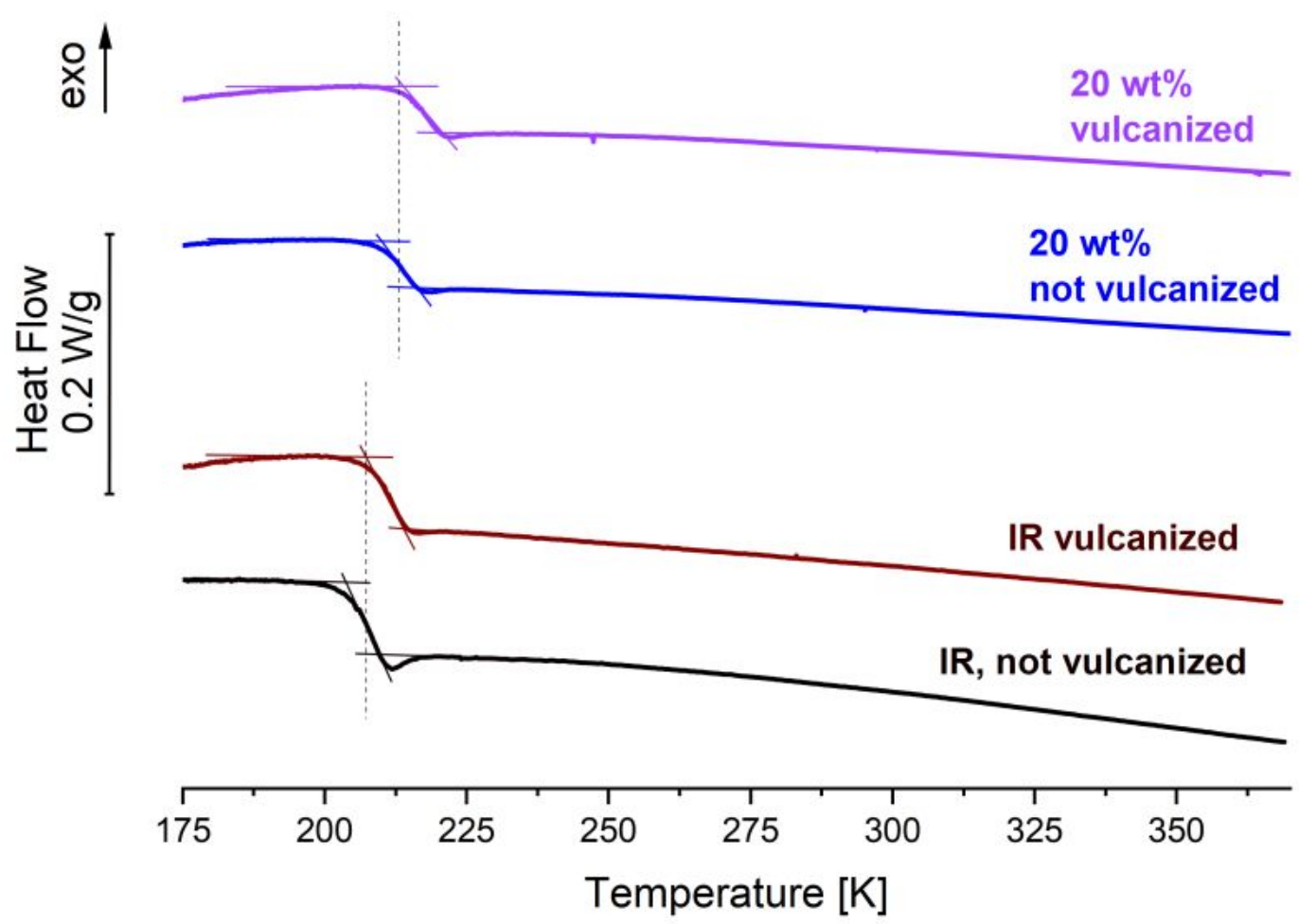

Figure S2: Heat Flow curves of IR and 20 wt% rosin ester in IR vulcanized by the ebonite method. In both cases, the glass transition temperature is shifted to higher values by approximately 3 K compared to the unvulcanized state, which is attributed to the restricted chain mobility resulting from sulfur crosslinking (see: Erman, B.; Mark, J. E.; Roland, C. M. *The Science and Technology of Rubber*, 4th edition.; Elsevier Academic Press: Amsterdam Boston, 2013.). Neither a separation of the glass transition nor a significant broadening is observed after vulcanization. This indicates that no additional phase separation or morphological rearrangement occurs during the ebonite process. Consequently, the nanoscale phase separation detected by TEM can be attributed to the inherent miscibility gap of the uncured blend rather than being an artifact of the vulcanization procedure.
